# COVID-19 Pandemic and Telephone Triage before Attending Medical Office: Problem or Opportunity?

**DOI:** 10.3390/medicina56050250

**Published:** 2020-05-20

**Authors:** Gabriele Cervino, Giacomo Oteri

**Affiliations:** Department of Biomedical and Dental Sciences and Morphological and Functional Imaging, Messina University, 1-98100 Messina, Italy; oterig@unime.it

**Keywords:** COVID-19, virus, epidemiology, infection risk, telemedicine

## Abstract

During the COVID-19 emergency, the medical operating protocols have been largely modified for reducing any type of contamination risk, for working in a safe way and for making the patient feel in a safe environment. Telemedicine, smart phones and apps could represent important devices for the community, in order to prevent virus trasmission and to perform quick diagnosis and management at medical offices. This manuscript could be useful for clinicians with regard to the current state of the effectiveness of the telephone triage in this COVID-19 epidemic period. Therefore, it could be an important starting point for future perspectives about telemedicine and virtual patient management.

In response to the COVID-19 pandemic, new prevention measures have been announced by governments around the world, measures that severely limit social interactions and travel, as well as visits at medical and dental offices.

According to guidelines, many clinicians and practitioners have changed their way of working, shifting to remote or smart working. While doctors, orthopedic surgeons, dentists and plastic surgeons have continued to provide non-deferred, urgent and emergency care, the restrictions pose a unique challenge for preventing contamination during the conventional front of house visits of many patients.

For this reason, the professional medical associations have worked to provide effective guidance on conducting telephone consultations, before visiting the patients. However, although this could be useful for controlling patient health status, medical and dental services cannot be based exclusively on remote assistance, because they are medical disciplines founded on experience and patient contact [1,2,3,4,5,6,7,8,9].

The professional and ethical conduct of the clinicians who carry out medical and dental care during this COVID-19 pandemic is a focus of attention in this period of global emergency. This pandemic is deeply changing the clinician’s medical habits, work attitudes and patient management [10,11,12,13,14].

COVID-19 is a respiratory virus that spreads mainly through contact with the respiratory droplets of infected people, direct mucous contact with saliva droplets or by mucous contact with those droplets on hands, on objects or on infected surfaces [1,4,13,14,15,16,17,18].

The dentist, working close to the patient and being in contact with saliva, is among the most exposed to the virus. However, daily dental practice has always been performed with personal protective equipment and with high attention to the hygiene standards of disinfection and sterilization [19,20,21].

Nevertheless, at this moment, the classification of the patients should be mandatory and should be carried out through preventive triage: a first evaluation summary of epidemiological data, including information about travel history, is mandatory. Then, the clinical presentation results reported by the patient himself are fundamental to assessing the probability of a coronavirus infection [22,23,24,25].

Triage prevention method gives clinicians the possibility of recognizing patients as potentially infected by coronavirus before accessing treatment [20,22,23,24,25,26].

The use of triage has been reported in the literature as effective in other areas, especially in the emergency medicine field and, therefore, reporting with the use of a questionnaire for the evaluation of symptoms in the context of primary care could be effective during the COVID-19 pandemic [14,18,20,21,22,23,24,25,26].

Triage consists of two phases: telephone and ’in-office’. Telephone triage makes it possible to evaluate the patient’s general health, identify the possible presence of symptoms related to COVID-19, as well as risky behaviors that may have increased the chances of the patient’s contact with infected subjects. There are two main reasons for concern regarding a second triage in office. Firstly, the predicted incubation period of COVID-19 makes it possible that the patient may have developed symptoms just before the clinical visit and after the phone call. Then, at the office, a second check records the patients as safe for being involved in the consequent medical treatments. It is also necessary to gather this data to protect clinicians and practitioners by a legal point of view too. The triages that took place in the studio must be signed and dated (with telephone triage data and in office triage data), and an appropriate questionnaire should be used. Triage is essential, because it represents an operative filter. Moreover, the patients’ cooperation in this situation is essential, as community health is important. The goal of patient examination is clinical decision, diagnosis and treatment; at this moment, the screening of COVID-19 risk patients and the accidental detection of asymptomatic pathologies still represent a challenge for clinicians [12,13,14,15,16,17,18,19,20,21,22,23,24,25,26,27,28].

At present, the consideration of a complete telemedicine system may seem premature, particularly in light of the general expectation that stricter social distancing measures will soon be relaxed. The deadlines are, however, indefinite, and the resumption of ’normal’ practice could still be hampered by the potential secondary peak in COVID-19 cases.

Currently, numerous smartphone applications, such as DOCTOROral® [28] diagnosis, are available for medical practitioners, clinicians and dentists, in order to perform presumptive quick diagnosis just by viewing a picture taken on the patient’s smartphone.

Remote consultations are essential for reducing the risk of infections. The operators should be invited to change their work habits to control the risk of contamination, and are asked to exercise their professional judgment to decide the level of post-therapy assistance and how to provide it.
